# Precision Medicine in Oral Health and Diseases: A Systematic Review

**DOI:** 10.3390/jpm13050725

**Published:** 2023-04-25

**Authors:** Giuseppina Malcangi, Assunta Patano, Mariafrancesca Guglielmo, Roberta Sardano, Giulia Palmieri, Chiara Di Pede, Elisabetta de Ruvo, Alessio Danilo Inchingolo, Antonio Mancini, Francesco Inchingolo, Ioana Roxana Bordea, Gianna Dipalma, Angelo Michele Inchingolo

**Affiliations:** 1 Department of Interdisciplinary Medicine, University of Bari “Aldo Moro”, 70121 Bari, Italy; assuntapatano@gmail.com (A.P.); m.guglielmo2@studenti.uniba.it (M.G.); robertasardano@gmail.com (R.S.); giuliapalmieri13@gmail.com (G.P.); c.dipede1@studenti.uniba.it (C.D.P.); studio.deruvo@libero.it (E.d.R.); ad.inchingolo@libero.it (A.D.I.); dr.antonio.mancini@gmail.com (A.M.); angeloinchingolo@gmail.com (A.M.I.); 2 Department of Oral Rehabilitation, Faculty of Dentistry, Iuliu Hațieganu University of Medicine and Pharmacy, 400012 Cluj-Napoca, Romania; roxana.bordea@ymail.com

**Keywords:** oral health, dentistry, precision medicine, genomics, oral diseases, metabolomic

## Abstract

Precision medicine (PM) is personalized medicine that can develop targeted medical therapies for the individual patient, in which “omics” sciences lead to an integration of data that leads to highly predictive models of the functioning of the individual biological system. They enable rapid diagnosis, assessment of disease dynamics, identification of targeted treatment protocols, and reduction of costs and psychological stress. “Precision dentistry” (DP) is one promising application that need further investigation; the purpose of this paper is therefore to give physicians an overview of the knowledge they need to enhance treatment planning and patient response to therapy. A systematic literature review was conducted on the PubMed, Scopus, and Web of Science databases by analyzing the articles examining the role of precision medicine in dentistry. PM aims to shed light on cancer prevention strategies, by identifying risk factors, and on malformations such as orofacial cleft. Another application is pain management by repurposing drugs created for other diseases to target biochemical mechanisms. The significant heritability of traits regulating bacterial colonization and local inflammatory responses is another result of genomic research, and is useful for DP in the field of caries and periodontitis. This approach may also be useful in the field of orthodontics and regenerative dentistry. The possibility of creating an international network of databases will lead to the diagnosis, prediction, and prevention of disease outbreaks, providing significant economic savings for the world’s health care systems.

## 1. Introduction

**Figure 1:**
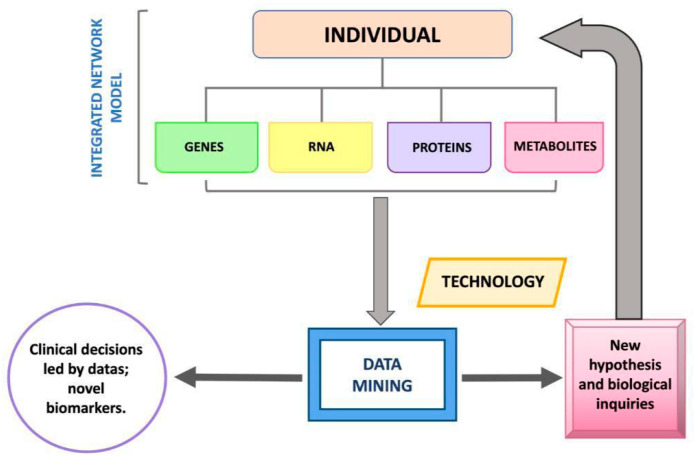
Illustrated process behind personalized medicine.

In recent decades, the development of high-throughput technologies has made it possible to develop rapid, reliable, and less expensive methods for complete DNA sequencing, so much so that it has led to a real revolution in genetic research, increasing its large-scale use [1]. Whole Genome Sequencing (WGS), or Whole Exome Sequencing (WES), i.e., sequencing of a coding nucleotide portion of the genome, qualitative and quantitative analysis of messenger RNA (transcriptome), analysis of the epigenetic influences of the genome that vary its expression (epigenome), and analysis of metabolites (metabolome) and proteins (proteome) in relation also to the eubiotic or dysbiotic environment (microbiome) are termed “omics” sciences [2]. The application of these technologies, implying a multidisciplinarity in skills and knowledge (encompassing engineering, medicine, physics, robotics, computer science, ethics, and biology) has generated precision medicine (PM) [3]. The term precision medicine (PM) indicates, therefore, personalized medicine capable of developing for the individual patient targeted medical therapies, in which, “omics” sciences (genomics, transcriptomics, epigenomics, proteomics, and metabolomics) lead to an integration of data leading to highly predictive models on the functioning of the individual biological system. They enable rapid diagnosis, assessment of disease dynamics by identifying targeted and effective treatment protocols, and reducing costs as well as psychological and physical stress [4,5] (Figure 1).

The emergence of PM associated with the complexity of “omics” sciences “catalyzes new organizational concepts in the Health Services system, compels investment in highly specialized personnel capable of using high-throughput technologies and producing, reading, and assembling big data derived from genomics [6].

PM is currently also being applied to oral cavity diseases and therefore referred to as “precision dentistry” (DP).

Particularly, DP is finding great use in the study of oral cavity tumors, which account for 50% of head–neck cancers [7,8,9].

DP has produced valuable findings regarding squamous cell carcinoma (SCC).

SCC accounts for 90% of oral carcinomas. Treatment is multidisciplinary: surgery, radiotherapy, and chemotherapy are often then accompanied by adverse outcomes, with patient death within five years of onset [10]. Although the early stage of SCC predisposes better than the advanced stage, there is still a mortality rate of 13% to 30%. The diagnosis appears to be closely related to perineural distance from SCC (“negative perineural invasion”) [11,12,13,14,15].

Chemotherapy (5-fluorouracil, carboplatin or cisplatin, taxol and irinotecan), as is used in the most severe and relapsing cases, always leads to side effects and death within a year of relapse because they no longer respond to therapy [14].

The presence of epithelial growth factor (EGFR) in SCC is consistently correlated with adverse outcome [16]. Hence, it was seen that combining radiotherapy with Cetuximab, a monoclonal antibody targeted against EGFR, resulted in greater control of SCC growth and spread [10,17,18,19].

**Figure 2:**
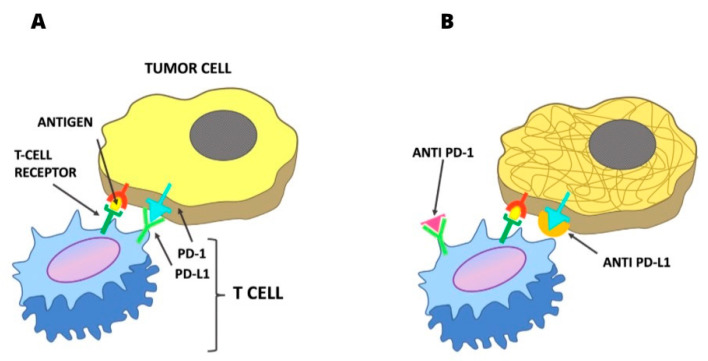
(**A**) Mechanism of inhibition of the PD-1/PD-L1 link on the antitumoral action of T lymphocytes. (**B**) Inibition PD-1 or PD-L1 link allowing the antitumoral action of T lymphocytes.

Through the data collected by The Cancer Genome Atlas on the oncogenome of oropharyngeal and oral cavity cancer, it was found that Phosphoinositide 3-kinase (PIK3CA) encoding the catalytic subunit PI3Kα is the most frequently mutated oncogene in oral cavity and oropharyngeal SCC (20%), and to a greater extent in human papillomavirus (HPV)-positive oropharyngeal SCC (25%) [6]. Oncogenomic analysis of SCC also revealed increased activation of the phosphatidylinositol 3-kinase/Akt/mammalian target of the rapamycin (PI3K/mTOR) pathway [20]. Inhibition of mTOR kinase, which regulates cell proliferation and controls DNA transcription and cell cycle progression, has antitumor effects in multiple experimental studies on oral cavity SCC. Metformin was found to be a good inhibitor of the PI3K/mTOR system [21] (Figure 2).

A special focus in the DP is on immunotherapeutics. The discovery of inhibition of T-cell antitumor activity by the PD-1 system through the PDL-1 ligand has directed studies toward immuno-oncotherapy [22].

In SCC, immunotherapeutic agents, such as PD-1-inhibiting pembrolizumab and nivolumab, have demonstrated, in a subgroup of patients with SCC of the oral cavity and oropharynx, a potent antitumor activity [23].

PM has given relevance to the study of the microbiome, which in addition to having metabolic functions for the proper maintenance of health in general or the onset of disease, may influence the metabolism of certain drugs and have an immunotherapeutic role [24,25,26]. The microbiomes of responder and non-responder patients to a given therapy could focus the study on pharmacodynamics for precision drug therapies [27,28]. The variability of the microbiome (MB) among individuals and the continuous mutability in the same individual by genetic and epigenetic factors requires crossing big data as well as high-definition technologies with rigorous informatics approaches [29,30,31]. PM can be applied in the field of the oral microbiome, where it can be used both for prevention and treatment in the strictly dental field (caries, periodontitis, cancers and orthodontics), and in systemic health and diseases of populations [32,33,34,35,36,37,38,39]. It is rich in biological and metabolic parameters, can help in evaluating balance and dysbiosis (dietary diets, lifestyle, immune system), and makes data analysis and collection easy.

The presence of biomarkers in saliva, such as cariogenic bacteria (mutans streptococci, Candida albicans, *Prevotella* spp.), histatin peptides, IgA, and IgG, is considered to predict caries risk [36,40,41]. Salivary biomarkers, such as salivary IgA and IgG against the pathogens Porphyromonas gingivalis and Treponema Denticola, salivary IgG against Aggregatibacter Actinomycetemcomitans, MIP-1a (Macrophage Inflammatory Protein-1a), interleukin 1β, (IL-1β), interleukin 6 (IL-6), Matrix metalloproteinase-8 (MMP-8), and Prostaglandin E2 (PGE2), are used to assess susceptibility to develop more or less severe periodontal diseases [42,43,44,45,46,47,48,49]. The presence of salivary insulin growth factor (IGF-1) detected by salivary proteomics can be used as a biomarker of skeletal growth, a relevant element for orthodontic treatment plans [42,50]. The use of blood products (Concentrated Growth Factor, CGF; Platelet-Rich Fibrin, PRF; Platelet-Rich Plasma, PRP) derived from venous sampling and applied in the regenerative therapy of mucosal and bone tissues of the same patient, and the use of MBP-1 and MBP-2 (bone morphogenetic proteins 1-2) in the regenerative bone of the oral cavity, derived from the trituration of the extracted tooth element of the patient using the Tooth Transformer device, are further examples of personalized and precision medicine [51,52,53,54,55,56,57,58].

**Figure 3:**
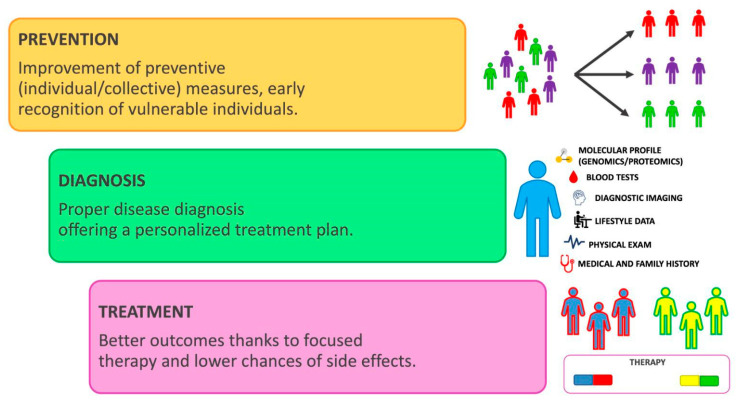
Schematic explanation of the personalized medicine model.

The investment of the Human Genome Project (HGP) also affected the ethical, legal, and social aspects of these scientific advances, introducing protection against genetic discrimination abuse (in employment, insurance, family, etc.) [59]. It should be considered that when a patient’s genetic data is utilized for research, therapeutic applications, or other purposes, their privacy should be protected. The possibility of creating an international database network will lead to diagnosing, predicting, and preventing the onset of rare diseases and cancers as well as establishing appropriate therapies, with the possibility also of significant economic savings for the global health care system [60]. The hypothesis is that the application of omics sciences, with the use of increasingly high-performance technologies in clinical diagnostics, will lead to preventive, personalized, and precision medicine with targeted, efficient therapies with fewer side effects. The purpose of this review is to give physicians an overview of the knowledge they need to enhance treatment planning and patient response to therapy in the field of precision dentistry (Figure 3).

## 2. Materials and Methods

### 2.1. Protocol and Registration

This systematic review was conducted according to Preferred Reporting Items for Systematic Reviews and Meta-Analyses (PRISMA), and the protocol was registered at PROSPERO under the ID: 413720.

### 2.2. Search Processing

A search on PubMed, Scopus, and Web of Science was performed to find papers that matched the topic of the influence of precision medicine and oral health, dating from 1 January 2013 to 1 March 2023. The search strategy used the Boolean keywords: “Genomics” AND (“oral health” OR “oral disease”) and “Precision Medicine” AND (“dentistry” OR “oral health” OR “oral disease”) (Table 1).

### 2.3. Inclusion Criteria

The following inclusion criteria were considered: (1) open access studies; (2) studies that investigated the relationship between precision medicine, genomics, and their implications in oral health; (3) randomized clinical trials, retrospective and observational studies; (4) English language; and (5) full text available.

Papers that did not match the above criteria were excluded.

The review was conducted using the PICOS criteria:

- Participants: adult patients, both male and female;
- Interventions: applications of precision medicine in dental practice;
- Comparisons: use of traditional medicine;
- Outcomes: Precision medicine can be useful in identifying prognostic factors;
- Study: randomized clinical trials, retrospective and observational studies.

### 2.4. Exclusion Criteria

The exclusion criteria were as follows: (1) animal studies; (2) in vitro studies; (3) off-topic; (4) reviews, case reports, case series, letters, or comments; (5) not written in English.

### 2.5. Data Processing

Three reviewers (M.G., R.S. and E.d.R.) independently consulted the databases to collect the studies and rated their quality, based on the selection criteria. The selected articles were downloaded into Zotero (version 6.0.15). Any divergence between the three authors was settled by a discussion with a senior reviewer (F.I.).

## 3. Results

### Study Selection and Characteristics

The electronic database search identified a total of 1782 articles (Scopus N = 194, PubMed N = 1441, Web of Science N = 147), and no articles were included through a search by hand.

**Figure 4:**
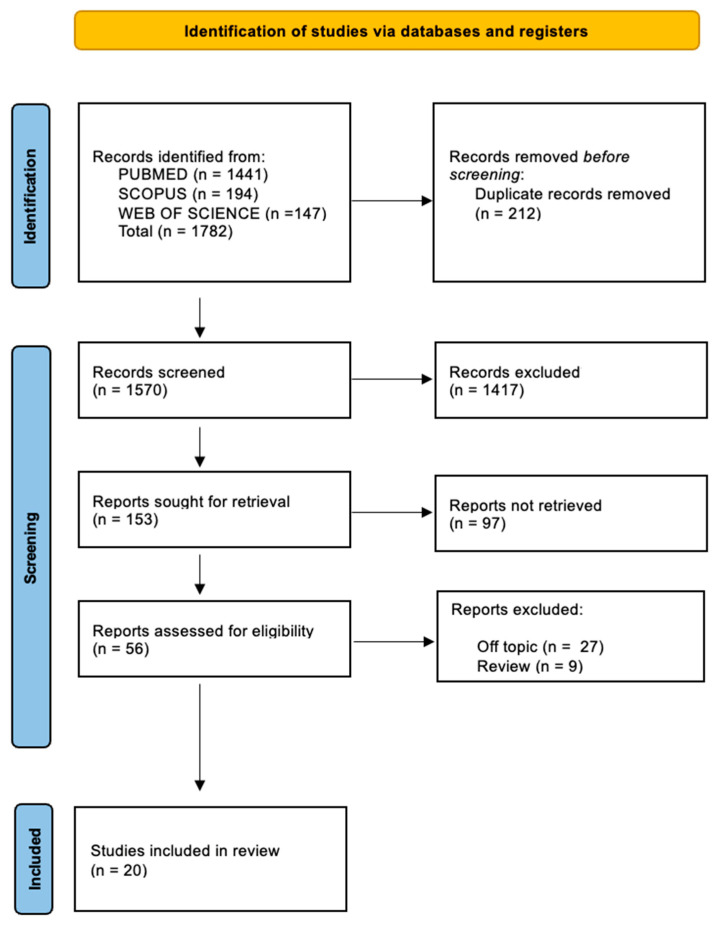
Literature search Preferred Reporting Items for Systematic Reviews and Meta-Analyses (PRISMA) flow diagram and database search indicators.

After the deletion of duplicates, 1570 studies were screened by evaluating the title and abstract, focusing on the association between precision medicine, genomics, and their implications in oral health. There were 1417 articles that did not meet the inclusion criteria (1206 off topic, 124 review, 87 animal studies), leading to 153 records being selected. Subsequently, 97 records were excluded (78 off topic, 12 reviews, 7 animal studies), and then 36 reports were excluded because they did not meet the inclusion criteria (27 off topic, 9 reviews). After eligibility, 20 records were selected for qualitative analysis. The selection process and the summary of selected records are shown in Figure 4 and Table 2, respectively.

## 4. Discussion

### 4.1. Precision Medicine and Strategies for Prevention and Treatment of Oral Cancers

#### 4.1.1. Squamous Cell Carcinoma

The oral cavity, the hypopharynx, the larynx, the nasopharynx, and the oropharynx are the sites most affected by cancer. Squamous cell carcinomas (SCC) account for at least 90% of neck and head cancer cases. The most frequently mutated oncogene in these carcinomas in general is PIK3CA, which encodes for the catalytic subunit PI3K [81].

PM aims to find effective therapies in the dental field as well, and genetic markers are being investigated that may allow personalizing the therapeutic approach toward various diseases, including cancers of the head and neck area; specifically, trial therapies toward squamous cell carcinomas (SCC) are already in place [77]. With the help of genomics, it is now possible to identify a number of potential targets for treatment for various cancers, but there is still a long way to go with regards to SCC. For this tumor type, one of the first targets identified in therapy was the receptor tyrosine kinase EGFR, and pairing it with radiotherapy or chemotherapy has shown encouraging results in controlling the disease [82].

In addition to focusing on treatment, PM is attempting to clarify cancer prevention options by identifying risk factors related to individuals. PM is personalized medicine, assessing not only an individual’s genetic characteristics, but also his or her personal and family history and the environmental factors to which he or she is exposed [63]. The best-known and most-studied risk factor is human papillomavirus (HPV); its correlation with the occurrence of some forms of oral and oropharyngeal cancer is well known, which is why vaccination against HPV has been promoted for years now. Other well-known risk factors include smoking and drug and alcohol use. The dentist, in clinical practice, has a great responsibility to intercept these risk factors and to raise awareness among patients to eliminate them where abuses are detected [63].

#### 4.1.2. Oral Tongue Squamous Cell Carcinoma

Oral tongue squamous cell carcinoma (OTSCC) is a very aggressive tumor, which easily develops loco-regional and distant metastases and recurs with high frequency; it has a low five-year survival rate regardless of the treatment approach taken. Currently, TNM staging is used to frame the severity and disease prognosis; however, this index does not consider many patient characteristics, for example, age, sex, and systemic inflammatory response. To develop a nomogram to predict survival after surgery based on clinical traits and serologic inflammation markers, Wei Lin et al. conducted a prospective study on a sample of 169 patients with this cancer. Specifically, significant prognostic factors proved to be the lymphocyte–monocyte ratio (LMN) and Immunoglobulin G (IgG) before therapy [61].

#### 4.1.3. Recurrent/Metastatic Carcinoma of Head and Neck

In recurrent/metastatic carcinomas of the head and neck region, Noji et al. considered whole-genome profiling and specifically evaluated whether it could have clinical value in identifying biomarkers that responded to immune checkpoint inhibitors. The top 30 most prevalent genetic mutations were examined in 54 patients. TP53 (62%), TERT promoter (56%), CDKN2A (21%), and PIK3CA (18%) were the most frequently mutated genes, including missense, nonsense, and splice site mutations, found in squamous cell carcinoma. Genes that were amplified frequently included Cyclin D1 (CCDN1), fibroblast growth factors, FGF3, FGF4, FGF19 (18%), EGFR (9%), and MYC (6%). In non-SCC cases, mutations in TP53 (30%), and deletions in CDKN2A/B (25%), and MTAP (20%) were found. Activating mutations or amplifications of ERBB2 and NTRK3 were discovered in 20% and 5% of cases, respectively, despite the absence of NTRK3 fusion. In contrast to forms with CCNDI1 amplification, which were less responsive and had a poor prognosis, it was found that forms with a high tumor mutational burden responded better to this type of approach [62]. According to recent studies, the human immune system can be a valuable ally in the battle against cancer. The body’s immune system can identify and eliminate cancer cells while preventing metastasis through the activation of T lymphocytes and other mechanisms.

Cancer cells use PD-1 as an “immune checkpoint”, or a method of evading immune surveillance. Cancer cells that express the two PD-1 ligands, PD-L1 and PD-L2, can inhibit immune responses by the PD-1 system.

The PD-1 receptor on T lymphocytes is bound by PD-L1 and PD-L2, inactivating them and enabling cancer cells to evade the immune response.

The predictive value of salivary biopsy in the early detection of head and neck cancer has also been studied. Cell-free DNA (cfDNA) and cell-free mitochondrial DNA (cf-mtDNA) can be isolated from saliva samples using a non-invasive, repeatable method. These markers are thought to be reliable indicators that can forecast the severity and prognosis of various cancers [64].

Saliva also was investigated in head–neck cancer patients to identify those at higher risk of developing oral mucositis associated with radiotherapy. Specifically, proteomics has shown that where there are certain proteins in higher concentration, there is a greater predisposition to have mucositis as an unwanted effect of radiotherapy [65].

Genomics is also useful in the prognostic evaluation of oral cancer, especially when information about the biology and molecular characteristics of tumors is correlated with clinical and pathological features [83].

#### 4.1.4. Poorly Differentiated Neuroendocrine Larynx Carcinoma

Laryngeal neuroendocrine carcinomas (LNECs) are uncommon, highly heterogeneous cancers that can manifest clinically and pathologically in a variety of ways. LNECs are uncommon, representing about 1% of all organ neoplasms. In today’s precision and personalized medicine, miRNA expression is essential for a quicker and more accurate diagnosis. Ricciardiello et al., in a clinical study from 2021, analyzed the miRNA expression profiles in laryngeal cancer tissues and discovered that miR-133b and miR-449a were significantly downregulated in comparison to their nearby normal counterparts. It’s interesting to note that miR-449a also demonstrated a potential for predicting the development of nodal metastases. Furthermore, they highlighted the diagnostic potential of miR-223, which was significantly upregulated in laryngeal tumor samples during the validation process. MicroRNAs serve as early, non-invasive, and potentially prescient biomarkers of chemotherapy response, and they represent a novel approach to diagnosis. Precision medicine has recently concentrated on the outstanding opportunities resulting from the potential to restore homeostatic miRNA levels to their therapeutic potential. This is tied to peculiar and specific patient profiles [69].

### 4.2. Precision Medicine and Orofacial Clefts

Orofacial clefts are a very large family of congenital deformities. Seventy percent of schisis cases are considered nonsyndromic. In the remaining cases, clefts fit into syndromic pictures (the most prevalent and best known syndromes are Pierre Robin syndrome and Van Der Woude syndrome) and are accompanied by other malformations: the eyes and cardiovascular and skeletal systems are the most affected sites. Newer genomic techniques, in combination with bioinformatics and statistical sciences, have enabled enormous advances in the etiopathogenesis of nonsyndromic forms. Since the 1990s, many risk genes and loci have been identified, first by linkage studies, which allowed the mapping of the Van Der Wouden syndrome locus. In 2002, mutations in the IRF6 gene were identified, and over the years this gene has been found to be associated with more than 350 mutations covering about 70% of Van Der Wouden syndromes. Due to genome-wide (GWAS) techniques, it has been discovered to be a crucial gene, implicated in non-syndromic forms as well. In addition to it, many other important genes have been identified.

Furthermore, epigenetic techniques are investigating another possible cause of disease: epi-mutations, that is, changes in DNA acetylation and methylation. However, this is a field that still requires more studies before it is fully understood [84].

Bartzela et al., in a retrospective study in 2021, sought to investigate transmission patterns by analyzing a very large sample of patients and their family members. The data collected are not easy to interpret, partly because there are environmental and additional factors to consider, such as race, gender, age of the parents, and mother’s exposure to smoking, alcohol, drugs, infections, and other potentially teratogenic agents during the early months of pregnancy. Their study found that bilateral forms of cleft lip-palate are more frequently associated with syndromes or other malformations and affect the male sex more. The female sex, in contrast, is more affected by cleft palate [66].

### 4.3. Precision Medicine and Cleidocranial Dysplasia

The skeletal condition known as cleidocranial dysplasia (CCD), which affects the clavicles, teeth, and sutures of the skull, is linked to RUNX2 mutations. Although many patients have been described, it has been challenging to establish a direct genotype–phenotype correlation for RUNX2.

Thaweesapphithak et al. conducted an interesting clinical study in 2022. The patients underwent clinical and radiographic examinations. Bioinformatics tools were used to analyze the data after exome and Sanger sequencing to identify genetic variants. Two cases were familial and three were sporadic. The heterozygous pathogenic RUNX2 variants were successfully identified by exome sequencing in all affected people. Three were new and two were almost known. The following uncommon or unreported phenotypes were seen in addition to the typical CCD features in all patients: left fourth ray brachymetatarsia, normal clavicles, phalangeal malformations, and normal primary dentition. Exome sequencing is effective at identifying mutations across ethnic groups, according to the study. The Runt homology domain is essential for RUNX2 function, as evidenced by the two p.Arg225 variants [71].

### 4.4. Precision Medicine Approach to Pain and Temporomandibular Disorders

Other methods for finding effective analgesics include those from precision medicine [85]. Repurposing medications created for other illnesses to target the biochemical mechanisms that control pain is one tactic. Adapting medication treatment based on patients’ genetics is another tactic [86]. The number of Americans who experience pain exceeds 100 million, and opioids are frequently given to treat pain [87]. The majority of opioids given in the US include tramadol, oxycodone, and codeine [87,88].

Codeine is bioactivated to morphine by the cytochrome P450 2D6 (CYP2D6) enzyme, while tramadol is bioactivated to O-desmethyltramadol, which has higher affinity for the opioid receptor than its parent molecules [88]. Certain isoforms of cytochrome P450 (CYP) play a central role in drug metabolism. Importantly, the presence of polymorphisms in these genes can greatly vary enzyme activity, resulting in phenotypes with different metabolic capacities. Genotyping can therefore provide insight into an individual’s response to a specific drug, and for some substrates of CYP2C9, CYP2C19, and CYP2D6 isoforms, individualization of therapy has become a reality for precision medicine [89].

Five to ten percent of people have no enzyme activity (PM, poor metabolizer), while another two to eleven percent have significantly decreased enzyme activity (IM, intermediate metabolizer). The ability of patients with CYP2D6 poor metabolizer or intermediate metabolizer phenotypes to biotransform codeine and tramadol into their active metabolites is decreased. Clinically, this might lead to inadequate analgesia, and findings indicate that these medications ought to be avoided in PMs and possibly in IMs [89].

Smith et al. conducted a study on the effectiveness of prescription opioids based on CYP2D6, demonstrating the success of this approach [67].

Thomas et al. evaluated the management of postsurgical pain based on CYP2D6 phenotype [79]. Good results were also achieved by Cavallari et al.: this trial encouraged the use of CYP2D6 genotyping in clinical practice, to be taken into account in conjunction with the use of CYP2D6 inhibitors, to guide customized opioid dosing to enhance postoperative pain control [78]. In the orofacial pain and Temporomandibular disorders (TMD), the use of PM needs great effort. A nonselective adrenergic receptor antagonist, propranolol, is effective for easing face pain and migraine [90].

There is evidence to suggest that variations in the catechol-O-methyltransferase (COMT) gene might affect the drug’s analgesic effectiveness. Slade et al. evaluated this hypothesis in an RCT study: the synergistic impact that was initially predicted was in direct opposition to the observed antagonistic effect of the A allele on propranolol’s effectiveness. If the COMT gene is to be exploited for precision medicine therapy of TMD, this surprising outcome underscores the need for deeper understanding of COMT’s function in pain causation [68]. However, difficulties remain and further investigations are needed, because the number of enzymes involved in drug metabolism is frequently large, and only for some are the clinical implications of polymorphism known.

### 4.5. Precision Medicine and Caries and Periodontitis

Although it has long been hypothesized that oral health is genetically determined, little is known about the heritable variation in the common characteristics of oral and dental illnesses that can be accounted for by the human genome [77]. Oral illnesses, such as dental caries, are significantly influenced by the microbiome found in the human mouth [80]. The passage of lactobacilli through the dentinal tubules and the presence of *Lactobacillus rhamnosus* in the pulp infection’s early stages suggest that lactobacilli play a significant role in the infection’s advanced phases [80]. Research was done on the invasion of living tooth pulp by the caries infection as it progressed through the dentin. Vital pulp tissue was infected in its early stages with *L. rhamnosus* [80,91].

In this study, the authors used high-throughput next-generation sequencing technology to learn more about the genomic characteristics of *L. rhamnosus* to identify any potential pathogenicity biomarkers [80]. The whole genomes of probiotic *L. rhamnosus* and tissue-invasive (obtained from infected dental pulps of decayed teeth) *L. rhamnosus* were compared. Category discrepancies were revealed through comparative genomic analysis [80].

By adherence to both cellular components and extracellular matrix, it is suggested that the surface aspects of clinical isolates of *L. rhamnosus* enable invasion of tooth pulp. By using the masking effect of an extracellular polysaccharide layer, such as the presence of capsular polysaccharide (CPS) or extracellular polysaccharide (EPS), an invasion could potentially be made easier [80,92]. As a result, it was shown that both clinical isolates’ altered EPS composition and pathogenicity were caused by the deletion of rmlACBD genes necessary for the dTDP-rhamnose biosynthesis pathway and the presence of a family 2 glycosyltransferase in the EPS cluster [80].

One of the most prevalent inflammatory illnesses affecting people, periodontal disease (PD) has a prevalence of 20 to 50% and affects 11.2% of people worldwide with severe periodontitis [93,94,95]. The phrase covers a wide range of pathological disorders, from mild gingival inflammation to the severe variety [95]. The accumulation of bacterial infections (bacterial plaque) is the primary etiologic factor [96,97,98,99]. Scaling and root planning (SRP), a nonsurgical mechanical and manual operation, is the standard therapy for periodontal diseases [100].

Ozone can lower the microbial load in the context of periodontal illness [101]. Its antiseptic effect is achieved by bacterial cell membrane rupture, which leads to bacterial lysis and death [102]. Ozone also interferes with regular cellular enzyme activity and lessens its effectiveness [103]. By modifying the breakdown of arachidonic acid-derived prostaglandins, which support the development of inflammation, the anti-inflammatory activity [104,105]. In this double-blind randomized clinical trial, it was seen that in the group treated with ozone in addition to SRP, there was an improvement in periodontal conditions compared with the group treated with SRP alone at 3 months [70]. There was a significant reduction in pocket probing in both groups [70].

The ASIC2 locus was found to be associated with severe gingival inflammation in the study of Agler et al. [77]. Another finding from genomic research was the substantial heritability of characteristics that control bacterial colonization and local inflammatory response. Therefore, for the purposes of this study, such features are potential candidates for genomic research [77].

To measure the contributions of genetic and environmental factors to the genesis of periodontal disease, a twin study was carried out [76]. It was discovered that hereditary variables accounted for 39% of tooth loss in men and 14% of tooth loss in women. Hence, a severe to moderate risk of periodontal disease is conferred by hereditary variables [76]. While non-shared factors accounted for the majority of the heterogeneity in periodontal disease, shared environmental factors—such as shared family experiences and habits—play an essential role in edentulism [76].

The goal of the study by Rakic et al. was to assess the diagnostic efficacy of osteoprotegerin (OPG) and nuclear factor kappa-B receptor activator (RANKL) in the diagnosis of peri-implantitis (PIMP) and peri-implant mucositis (PIM) [75]. Bone resorption was present in PIM, as evidenced by bone turnover markers (BTMs) [75]. The ability of BTMs to significantly enhance clinical diagnosis of peri-implant diseases has been shown [75].

### 4.6. Precision Medicine and Growth Factor

A growth factor is a bioactive protein implicated in stimulated cell proliferation, wound healing and occasionally cellular differentiation [106].

Many resources of growth factors such as PRP, PRF, CGF, and individual patient’s blood products, are widely used for the process of bone and soft tissue regeneration in dentistry.

Although PM is still in development and many working mechanisms of growth factors remain unclear [107], growth factors play an important role in the concept of personalized therapy and many studies have demonstrated that it can be an important aid in regeneration medicine. In fact, in a previous single-blind clinical trial work, the wound healing index revealed a statistically significant difference (*p* = 0.001) in the test sides with the placement of CGF compared to the control sides left to heal naturally. The use of CGF improves the patient’s post-operative outcome helping wound stability.

In osteogenic and odontogenic regeneration dentistry, growth factors also provide an important antibacterial/pro-angiogenic therapeutic effect. The use of the Vascular Endothelial Growth Factor (VEGF) combined with Cu-BGn showed great advantages in a rat molar tooth defect infected with *E. faecalis.* The antibacterial properties against one of the most common pathogenic bacteria causing odontogenic infections and the capacity of up-regulation of VEGF stimulating angiogenesis of endothelial cells open new possibilities for the application of nanotherapeutic approaches in precision medicine [108].

### 4.7. Precision Medicine and Orthodontics

Patient-based treatments can be helpful in the field of orthodontics, notwithstanding the complex interaction between genetic and non-genetic variables. 

One of the major risks in orthodontic treatment is orthodontically induced external apical root resorption (OIEARR). An observational study on 195 patients requiring orthodontic treatment analyzed nine clinical and treatment variables, along with single nucleotide polymorphisms (SNPs) from five genes and variable interactions as risk factors for OIEARR using a multiple linear regression model. This study highlights the fact that interactions between genetic and non-genetic factors are dynamic and evolves over time. For this reason, genetic testing cannot prevent this iatrogenic event [73]. 

Another work dealing with the effect of supplemental vibratory force on biomarkers of bone remodeling during orthodontic tooth movement and the rate of mandibular anterior alignment (RMAA) examines the concentration of selected biomarkers of bone remodeling with salivary multiplex assay before treatment (T0) and at three following time points (T1, T2, T3). No correlation in the changes in salivary biomarkers of bone remodeling and RMAA is detected with supplemental vibratory force during orthodontic treatment with fixed appliances [72].

## 5. Conclusions

Precision medicine is a modern, comprehensive, data-driven method that divides comparable patients into phenotypic groups based on individual features.

PM is also attempting to shed light on cancer prevention strategies and on etiopathogenesis of malformations, such as orofacial cleft.

Another application of precision medicine is the management of pain to target biochemical mechanisms or adapting treatment based on patients’ genetics.

The significant heritability of traits that regulate bacterial colonization and local inflammatory responses was another discovery from genomic research, useful for precision dentistry in the field of dental caries and periodontitis.

Despite the complicated interactions between genetic and non-genetic factors, this patient-centered approach can also be useful in orthodontics and regenerative dentistry. The possibility of creating an international database network will lead to diagnosing, predicting, and preventing the occurrence of rare diseases and cancers, as well as establishing appropriate therapies, with the possibility also of significant economic savings for the world health care system. Precision dentistry requires the implementation of novel clinical trial strategies to become more relevant. In fact, in the field of dentistry, along with the use of a very large set of patient data, PM is initiating towards a less interventionist medicine, which can guide the reformulation of treatment protocols, orienting them more and more towards a preventive, diagnostic, and therapeutic personalized approach. The possibility of realizing personalized programs that can monitor the microbial environment of the oral cavity responsible for caries, periodontitis, and cancers, in addition to masticatory (temporomandibular joint dysfunction) and respiratory (e.g., obstructive sleep apnea syndrome) function would lead to a high degree of predictability with a high cost–benefit ratio both biological and economic.

## Figures and Tables

**Table 1:** Database search indicators.

Articles screening strategy	KEYWORDS: A: genomics; B: precision medicine; C: oral health; D: oral disease; E: dentistry
	Boolean Indicators: A AND (C OR D) B AND (E OR C OR D)
	Timespan: 2013–2023
	Electronic databases: PubMed; Scopus; WOS

**Table 2:** Descriptive summary of item selection.

Authors	Study Type	Aim of the Study	Number of Patients	Gender and Average Age	Materials and Methods	Results
Lin et al., 2021 [61]	Retrospective study	The purpose of this study was to develop a nomogram to evaluate oral tongue squamous cells carcinoma patients’ post-operative outcomes	169 patients with pathologically proven oral tongue squamous cell carcinoma.	The median age for patients was 57 years (range 25–88 years), of which 93 (55%) were males and 76 (45%) were females.	Analysis was carried out to identify the independent prognostic indicators linked to the patient’s overall survival.. Using a bootstrap resampling technique, a nomogram based on these predictive factors was created and internally validated.	The results of time-dependent C-index for overall survival also showed that the nomogram had a better discriminative ability than that of tumor/node/metastasis stage. The calibration curves of the nomogram showed good consistency between the probabilities and observed values.
Noji et al., 2022 [62]	Randomized clinical trial	The study’s objective was to assess the relationships between multiple predictor candidates, such as tumor mutational burden, programmed cell death ligand-1 expression rates, and gene alterations and immune checkpoint inhibitor response in patients with head and neck squamous cell carcinoma.	1119 patients diagnosed with recurrent/metastatic head and neck cancer.	The median age was 61 years, of which 55% were female and 45% male.	Cases of head and neck cancer were evaluated using the nationwide genetic clinical database established by the Center for Cancer Genomics and Advanced Therapeutics and 54 cases in an institution-based study.	The results indicate that comprehensive genomic profiling may be useful in identifying prognostic biomarkers for immunotherapy in patients with head and neck cancer.
Chang et al., 2013 [63]	Randomized clinical trial	This study’s objective is to use a combination of feature selection and machine learning techniques to predict the prognosis of oral cancer using the parameters of the association between clinicopathologic and genomic markers.	31 oral cancer cases were selected from the Malaysian Oral Cancer Database.		Five feature selection approaches have been proposed and tested on the oral cancer prognostic dataset during the initial phase of this project. The model with the features chosen from each feature selection method is evaluated on the suggested classifiers in the second stage.	The findings showed that the prognosis is better when both clinicopathologic and genetic markers are present. To confirm the potential of the chosen traits to serve as a significant prognostic signature in oral cancer studies, more research can be done on them.
Sayal et al., 2022 [64]	Randomized clinical trial	The purpose of this study was to evaluate the effectiveness of saliva-based liquid biopsy as a non-invasive source of cell-free DNA and cell-free mitochondrial DNA for identifying head and neck cancer.	137 healthy participants and 133 patients with either oral leukoplakia or head and neck cancer were compared.	The median age was 48 years, of which 60% were male and 40% female.	Each participant provided a sample of entire, unstimulated saliva. Using multiplex quantitative PCR, the absolute copy numbers of cell-free and mitochondrial DNA in saliva were measured. The ability of two diagnostic indices based on the molecules under investigation to distinguish between various diagnostic categories was evaluated.	Patients with severe epithelial dysplasia had a median score of cell-free DNA that was statistically greater than those with mild epithelial dysplasia and epithelial keratosis without severe epithelial dysplasia. The ability to employ cell-free DNA and cell-free mitochondrial DNA as diagnostic tools for head and neck cancer was demonstrated.
Jehmlich et al., 2016 [65]	Prospective study	The full proteome dataset (raw and search files) of patients at baseline for radiotherapy treatment, as determined by nano liquid chromatography coupled to mass spectrometry, is provided in this dataset.	50 patients before receiving radiotherapy.	Unspecified.	The full proteome dataset (raw and search files) of patients at baseline for radiotherapy treatment, as determined by nano-liquid chromatography coupled to mass spectrometry, is provided in this dataset by the searchers. 5323 tryptic peptides that belong to 487 different proteins were found in the data set.	After therapy, oral mucositis (grade III) occurred in 41 of 50 patients, with 14 of these patients displaying early oral mucositis (grade III) with a low radiation dosage of 30 Gy. Oral mucositis did not spread to nine patients (grade III).
Bartzela et al., 2021 [66]	Observational study	The study’s objective is to clinically describe patients and family members with cleft lip and/or palate and related congenital disorders or abnormalities, and to suggest potential inheritance patterns.	266 index patients with cleft lip and/or palate had accepted to participate.	Unspecified.	Added to the patient’s medical and family history was a standard questionnaire that was tailored to the demands of the study (SI 1). The questionnaire was fully completed by the patients. A clinical geneticist provided confirmation of the diagnosis in cases of suspected syndromes.	There were 24 (9.2%) syndromes and 74 (27.8%) related malformations in the individuals. The most often affected systems were the skeletal (27.7%), cardiovascular (19.3%), and eye (22.9%) systems. The most prevalent disorders were van der Woude (4) and the Pierre Robin Sequence (7 individuals). Nineteen out of twenty-four patients (19/24) had an associated syndrome.
Smith et al., 2019 [67]	Non-randomized clinical trial	The effects of prescription opioids based on CYP2D6 on pain management were investigated in this study. Intermediate and poor metabolizers (Ims and PMs) are predicted to have decreased analgesia because CYP2D6 bioactivates codeine and tramadol.	375 patients were enrolled, 239 assigned to CYP2D6-guided care, 136 assigned to usual care.	The median age was 59 years, of which 66% were male and 34% female.	Using a cluster design, participants with chronic pain (94% on an opioid) were enrolled into the CYP2D6-guided or usual care arms. These participants came from 7 clinics. Recommendations for prescribing opioids were generated in the CYP2D6-guided arm after CYP2D6 phenotypes were allocated based on genotype and the usage of CYP2D6 inhibitors. PROMIS^®^ measurements were used to evaluate pain at baseline and three months.	Intermediate and poor metabolizers who were first prescribed tramadol/codeine (n = 45) experienced greater improvement in the CYP2D6-guided versus usual care arm (1.011.59 versus 0.401.20; adj-*p* = 0.016); 24% of CYP2D6-guided participants compared to 0% of usual care participants reported 30% (clinically meaningful) reduction in the composite outcome.
Slade et al., 2021 [68]	Randomized clinical trial	The purpose of the study was to examine if, in a subset of individuals with painful TMJ condition, catechol-O-methyltransferase gene variations would affect analgesic efficacy.	200 adults.	Adults aged 18 to 65 y with TMD myalgia (with or without arthralgia).	Participants were randomly assigned to receive propranolol 60 mg twice daily or a placebo after being genotyped for the COMT single nucleotide polymorphism rs4680. Patients provided daily ratings of the severity and duration of their facial pain during the 9-week follow-up period; the product was then calculated as an index of facial pain.	While the dichotomized 30% decrease demonstrated stronger efficiency among G:G homozygotes than among A:A homozygotes with statistically significant interaction, the mean index reduction did not differ substantially (*p* = 0.277) according to genotype. Greater efficacy for G:G homozygotes than for A:A homozygotes was demonstrated by cumulative response curves. The synergistic impact that was initially predicted was in direct opposition to the observed antagonistic effect of the A allele on propranolol’s effectiveness. If the catechol-O-methyltransferase gene is to be exploited for precision medicine therapy of TMJ dysfunction, this surprising result emphasizes the need for greater understanding of the role of catechol-O-methyltransferase in pain pathogenesis.
Ricciardiello et al., 2019 [69]	Randomized clinical trial	This study’s objective is to report on experience with a group of patients who have laryngeal neuroendocrine carcinomas in order to identify clinical characteristics, treatment outcomes, follow-up, overall survival, and microRNA profiling to suggest a diagnostic–therapeutic signature.	10 consecutive cases of laryngeal neuroendocrine carcinoma.	Adults aged 48 to 74 years; 7 male and 3 female.	Patients enrolled in the study underwent Ear–Nose–Throat examination by laryngoscopy, neck, and intravenous contrast-enhanced CT scan, a biopsy performed during direct microlaryngoscopy, and a histological examination to confirm the diagnosis of laryngeal neuroendocrine carcinomas. A microRNA study that revealed a distinctive signature likely connected to poorly differentiated larynx neuroendocrine carcinomas was evaluated and compared with clinical characteristics with PCR test.	Poorly differentiated Laryngeal neuroendocrine carcinomas should have a special characteristic called a microRNA signature, which would justify further validation on a larger cohort.
Rapone et al., 2022 [70]	Randomized clinical trial	The study’s hypothesis is that adding gaseous ozone therapy to routine periodontal care may help to stimulate the body’s natural healing process.	90 healthy individuals with moderate or severe generalized periodontitis.	Unspecified.		
Thaweesapphithak et al., 2022 [71]	Clinical study	To identify clinical variants of Cleidocranial Dysplasia.	Five patients with Cleidocranial Dysplasia and their relatives.	5 patients: 3 sporadic cases and 2 familiar.	Exone sequencing technique.	A new feature was found (unilateral brachymetatarsia) and 3 new truncating variants of RUNX2.
Reiss et al., 2020 [72]	Clinical study	Evaluate influence of vibration devices on bone remodeling.	40 patients.	Age between 15 and 35 years.	Salivary test to analyze salivary biomarkers before treatment and 4–6 weeks later for 3 successive detections.	No influence on biomarkers of bone remodeling.
Silva et al., 2022 [73]	Clinical study	Assess factors influencing susceptibility to orthodontically induced external apex root resorption.	195 patients.	Unspecified.	Multiple linear regression model to analyze 9 clinical variables, single nucleotide polymorphisms of 5 genes, and variable interactions.	The impact of genetics is variable according to clinical forms.
Elayah et al., 2022 [74]	Randomized clinical study	Evaluate effect of concentrated growth factor on postoperative lower third molar extraction included.	37 patients.	Unspecified.	Patients undergoing bilateral extraction, 1 site treated with CGF and the other not, and evaluation of postoperative sequelae at 1–3–7 days.	Application of CGF at the surgical site reduces postoperative sequelae in terms of pain and swelling.
Rakic et al., 2020 [75]	Clinical study	Developing a personalized diagnostic model to monitor implant health.	126 patients and 252 implants.	Unspecified.	Assessed concentration of RANK (nuclear factor kappa-B receptor activator) and OPG (osteoprotegerin) in peri-implant crevicular fluid, along with evaluation of some clinical parameters.	Markers of bone turnover can be useful in diagnosing implant health but must be correlated with clinical values.
Mucci et al., 2005 [76]	Retrospective study	Assess influence of environmental factors and genetic factors for periodontal disease.	10,000 pairs of Swedish twins.	Unspecified.	Information obtained by telephone interviews and then processed with statistical tests.	Genetic factors influence 14% tooth loss, nonshared environmental factors 25%, shared environmental factors remaining risk.
Agler et al., 2019 [77]	Cohort study	Genome-wide GWAS technology to identify genetic factors in the development of periodontal disease and caries.	40,000 adults.	Unspecified.	Genetic analysis and clinical evaluation correlated with statistical analysis of results.	Biologically defined traits are more heritable than clinically defined traits.
Cavallari et al., 2022 [78]	Randomized clinical study	Evaluate effect of CYP2D6 (cytochrome P450 2D6 enzyme) administration in controlling postoperative pain.	Ongoing patient enrollment until June 2023.	Age 8 years or older.	Pain assessment at 10 days, 1–3–6 months after surgery by questionnaire.	The study will provide data on the clinical utility of CYP2D6-driven opioid selection.
Thomas et al., 2021 [79]	Randomized clinical study	Evaluate effect of CYP2D6 administration in the control of postoperative pain.	260 patients.	Adults over the age of 18.	Patients undergoing total joint arthroplasty, divided into groups. Test group: genotype-driven approach to pain. Control group: traditional pain approach.	Use of CYP2D6 helpful in controlling postoperative pain.
Nadkarni et al., 2014 [80]		In this study, researchers aimed to investigate the genomic characteristics of tissue-invasive *L. rhamnosus* and identify biomarkers that could reveal information about the pathogenic potential of lactobacilli, which are typically thought to be safe.	From the dental pulp-invasive *L. rhamnosus* strains LRHMDP2 and LRHMDP3 isolates, the study discovered 264 and 258 genes, respectively.	Unspecified.	The complete genome sequences of two clinical isolates of *L. rhamnosus* infecting viable pulp were generated using Roche GS FLX+ technology.	Genomic distance analysis and SNP divergence confirmed a close relationship between clinical isolates and segregation from the reference probiotic strain *L. rhamnosus*.

## Data Availability

Not applicable.

## Abbreviations

BTMs	Bone Turnover Markers
CCD	cleidocranial dysplasia
cfDNA	Cell-free DNA
cf-mtDNA	cell-free mitochondrial DNA
CGF	Concentrated Growth Factor
CCND1	Cyclin D1
CYP	cytochrome P450
COMT	catechol-O-methyltransferase
CPS	Capsular Polysaccharide
DNA	Deoxyribonucleic Acid
DP	precision dentistry
EGFR	receptor tyrosine kinase
EPS	Extracellular Polysaccharide
GWAS	genome-wide techniques
HGP	Human Genome Project
HPV	Human Papillomavirus
IGF-1	insulin growth factor
IgG	Immunoglobulin G
IL-1β	interleukin 1β
IL-6	interleukin 6
LMN	lymphocyte-monocyte ratio
LNECs	Laryngeal neuroendocrine carcinomas
MB	microbiome
MMP-8	Matrix metalloproteinase-8
OIEARR	Orthodontically Induced External Apical Root Resorption
OPG	Osteoprotegerin
OTSCC	Oral tongue squamous cell carcinoma
PD	Periodontal Disease
PGE2	Prostaglandin E2
PIM	Peri-implant Mucositis
PIMP	Peri-implantitis
PM	Precision Medicine
PRF	Platelet-Rich Fibrin
PRP	Platelet-Rich Plasma
RANKL	Nuclear Factor Kappa-B Receptor Activator
RMAA	rate of mandibular anterior alignment
RNA	Ribonucleic Acid
SCC	Squamous-cell Carcinoma
SNPs	Single Nucleotide Polymorphisms
SRP	Scaling and Root Planning
WES	Whole Exome Sequencing
WGS	Whole Genome Sequencing
VEGF	Vascular Endothelial Growth Factor
